# A tale of two STs: molecular and clinical epidemiology of MRSA t304 in Norway 2008–2016

**DOI:** 10.1007/s10096-021-04353-9

**Published:** 2021-10-23

**Authors:** Hege Enger, Kjersti Wik Larssen, Elise Størvold Damås, Hege Vangstein Aamot, Anita Blomfeldt, Petter Elstrøm, Christina Gabrielsen Ås

**Affiliations:** 1 The Norwegian MRSA Reference Laboratory, Department of Medical Microbiology, St. Olavs Hospital, Trondheim University Hospital, Trondheim, Norway; 2 Department of Microbiology and Infection Control, Akershus University Hospital, Lørenskog, Norway; 3 Department of Antibiotic Resistance and Infection Prevention, Norwegian Institute of Public Health, Oslo, Norway; 4 Department of Clinical and Molecular Medicine, Norwegian University of Science and Technology, Trondheim, Norway

**Keywords:** MRSA, t304/ST6, t304/ST8, Surveillance, Genotyping, Epidemiology

## Abstract

The purpose of this study was to investigate the epidemiological, molecular, and clinical characteristics of MRSA t304/ST8 and t304/ST6 in Norway from 2008 to 2016. Clinical and epidemiological data were collected for each case included in the study. Strains were characterized by PCR, *spa* typing, antimicrobial susceptibility testing, and whole genome sequencing. The overall number of cases of MRSA t304 increased from 27 in 2008 to 203 in 2016. Most MRSA t304/ST8 cases were defined as HA-MRSA (89.9%) and diagnosed in persons with Norwegian background, many of them living in nursing homes (62.3%). The number of t304/ST8 cases declined throughout the study period and it has not been reported in Norway since 2014. The increasing MRSA t304/ST6 genotype has mainly been introduced to Norway by immigration from the Middle East, but also from other parts of the world. The t304/ST6 clone is mostly classified as CA-MRSA (75.1%), does not seem to cause serious infections, is not multi-resistant, and has not yet caused outbreaks in Norway. This study provides an example of two MRSA clones with the same *spa* type found in different epidemiological settings. This is very unusual, but still a reminder that *spa* typing in some cases may have insufficient discriminatory power for surveillance of MRSA. Our results highlight the importance of active surveillance and characterization of emerging MRSA clones with high potential for spread in the community, which may potentially cause outbreaks in healthcare facilities.

**Supplementary Information:**

The online version contains supplementary material available at 10.1007/s10096-021-04353-9.

## Introduction

*Staphylococcus aureus* causes a wide spectrum of infections ranging from asymptomatic carriage to skin and soft tissue infections, bone and joint infections, bacteraemia, and endocarditis. Methicillin-resistant *S. aureus* (MRSA) possesses resistance against the bactericidal beta-lactam group of antibiotics, which is the cornerstone of treatment for staphylococcal infections. The incidence of MRSA varies in different parts of the world, but the total disease burden and mortality is considerable. A European study estimated a total of 33,000 deaths due to infections with resistant microbes in the EU and the European Economic Area in 2015, with MRSA contributing to a substantial part of the mortality and morbidity [1].

The incidence of MRSA in Norway is low compared to many other countries, with 49 per 100,000 person years in 2016 [2]. The proportion of MRSA in both *S. aureus* bloodstream infections (BSI) and wound infections has been stable at around 1% the last decade in Norway [2, 3]. However, from 2008 to 2016, the total number of reported MRSA infections and carriage strains in Norway has gradually increased from 652 to 2538 cases per year [2, 3]. One of the genotypes that has contributed most to this increase, both in Norway and in the other Nordic countries, is MRSA *spa* type t304 [4]. MRSA t304/ST8 was reported as the cause of several nursing home-related outbreaks in South East Norway from 2005 to 2011 [5]. This genotype has also been described in Martinique in the Caribbean [6].

In 2015, a study describing a prolonged neonatal ward-related outbreak in Copenhagen caused by MRSA t304/ST6 was published [7], and the same year, this genotype was detected by the Norwegian MRSA reference laboratory. MRSA t304/ST6 had previously been reported as a dominating genotype in Oman [8], and had been detected in the United Arab Emirates [9], as well as in *cfr*-positive MRSA strains from pigs in China [10].

In this study, we wanted to explore and compare these two genotypes (MRSA t304/ST6 and t304/ST8) with regard to epidemiological, molecular, and clinical characteristics, in an attempt to elucidate the origins of these genotypes, possible adaptations to healthcare and community settings, and whether MRSA t304/ST6 may represent a high risk clone that is becoming dominant in Norway.

## Materials and Methods

### Study design and population

All laboratory-confirmed cases of MRSA in Norway, including both carriage and infections, are notifiable to the Norwegian Surveillance System for Communicable Diseases (MSIS), and all new MRSA strains are sent to the Norwegian MRSA reference laboratory for confirmation, genotyping, and biobank storage. All cases of MRSA t304 in Norway from 2008 to 2016 were included, one strain per individual that had given passive informed consent to participate in the study. After exclusion of persons without available contact information (*n* = 79), 318 cases were included in the study.

### Epidemiological data

Epidemiological data on all included cases were collected from the Norwegian Surveillance System for Communicable Diseases (MSIS), the Norwegian National Population Registry, the Norwegian Healthcare worker registry (HPR), and from the requisition filled out by the treating physicians. The HPR contains information about all persons educated as healthcare workers (HCWs). Information from MSIS includes age, sex, birth country of the person and the person’s parents, admission to hospital or nursing home, and travel history, in addition to information about the bacterial strain. In this study, due to lack of data on admission time for hospitalized patients, we used a broad definition of healthcare-associated MRSA (HA-MRSA). This included MRSA diagnosed in hospitals or nursing homes and/or MRSA diagnosed in HCWs, while community-acquired MRSA (CA-MRSA) was defined as all other cases. Statistical analyses of discrete variables were performed using Fisher’s exact test, while age distributions were compared with Welch’s *t*-test. The Benjamini–Hochberg method was used to correct for multiple hypothesis testing, with adjusted *p*-values < 0.05 regarded as statistically significant.

### Bacterial strains and antibiotic susceptibility testing

Bacterial strains were cultured on blood agar plates at 35 °C, if poor growth incubated with 5% CO_2_. Susceptibility testing was performed on all strains using the EUCAST (European Committee on Antimicrobial Susceptibility Testing) disk diffusion method and categorized as either susceptible, intermediate, or resistant according to the 2016 EUCAST breakpoints. For clindamycin, inducible resistance was recorded as described in the EUCAST expert rules. For vancomycin, gradient strip test was used. For strains from 2008 to 2015, susceptibility testing was performed by the reference laboratory using disks from Oxoid and gradient strip tests from Biomerieux and Liofilchem. From 2016, susceptibility testing was performed by the referring laboratories, and results were reported to the reference laboratory.

### DNA extraction, confirmation PCR and *spa* typing

Extraction of genomic DNA was routinely performed by heat lysis. Briefly, a few colonies from pure culture were suspended in molecular grade water and heated to 95 °C for 15 min with shaking (300 rpm). After centrifugation at 14,500 rpm for 2 min, the supernatant was extracted and used for PCR analyses.

Confirmation of all MRSA strains was performed using a multiplex conventional PCR detecting the *mecA* gene, the *S. aureus*-specific *spa* gene, and the Panton-Valentine leukocidin (PVL) genes followed by gel electrophoresis [11]**.**

All received strains were genotyped by *spa* typing, sequencing of the polymorphic region X of the *S. aureus* specific *spa* gene according to Harmsen et al. [12] using primers *spa*-1113f and *spa*-1514r [13]. The sequences obtained were assigned to *spa* types using the Ridom *Spa* Server [14].

### Discriminatory high-resolution melt-PCR

To enable rapid differentiation between MRSA t304/ST6 and t304/ST8, a high-resolution melt-PCR (HRM-PCR) was established based on a discriminatory single nucleotide polymorphism (SNP) in position 294 the phosphate acetyltransferase (*pta*) allele. Real-time PCR was performed with forward and reverse primers (5′-AAGCAGATGGTTTAGTTAGT-3′ and 5′-ATACACCTGGTTTCGTTT-3′ accordingly) by a two-step PCR program on a CFX96 Real-time system (Bio-Rad). HRM analysis was performed using the Precision Melt Analysis™ Software.

### Whole genome sequencing and bioinformatics analyses

Whole genome sequencing (WGS) was performed on a subset of randomly selected strains from each year of the study period, maximum one strain from each household, including 20 t304/ST8 and 88 t304/ST6. This covered 43.9% and 48.6% of all households of ST8 and ST6 cases accordingly.

For WGS, cells were first treated with proteinase K (2 mg/mL) and lysostaphin (0.1 mg/mL) for 15 min with shaking at 37 °C, before heating for 15 min at 65 °C. Genomic DNA was then isolated using the EZ1 DNA tissue kit with an EZ1 Advanced XL instrument (Qiagen). Sequencing libraries were prepared using the Nextera XT sample prep kit and sequenced on the MiSeq platform with MiSeq v3 reagents, with 300 bp paired end reads (Illumina).

Raw data were quality controlled, trimmed/filtered, and de novo assembled, and the assembled genomes annotated, typed (multi-locus sequence typing (MLST)), and characterized (resistance and virulence genes) with the Nullarbor pipeline version 2.0 [15]. Resistance and virulence genes were identified using the NCBI National Database of Antibiotic Resistant Organisms (NDARO) [16] and the Virulence Factor DataBase (VFDB) accordingly [17]. Additionally, SCC*mec*Finder 1.2 was used for SCC*mec* typing of all strains [18]. The core and accessory genome of the study strains and 15 *S. aureus* reference genomes was defined and a core genome alignment produced by Roary version 3.13 [19]. Fasttree 2.1.10 [20] was used to infer a maximum likelihood core genome phylogeny with the GTR model, and distance estimation was performed by Molecular Evolutionary Genetics Analysis (MEGA) software [21]. Genetic distances in general refer to the number of SNPs in a *S. aureus* core genome alignment of 1,280,334 bp. Visualizations of phylogenies with metadata were created using iTOL [22].

## Results

### Temporal distribution

**Fig. 1:**
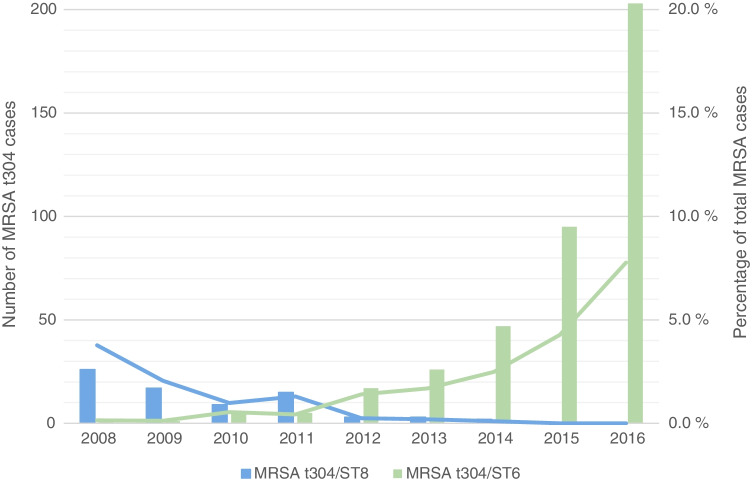
Temporal distribution of MRSA t304/ST8 (*n* = 75) and t304/ST6 (*n* = 400) cases (bars) and as a percentage of total MRSA cases in Norway (lines)

A total of 475 MRSA t304 strains were received by the Norwegian MRSA reference laboratory in the 9 year study period (2008–2016), with an overall increase in cases from 27 in 2008 to 203 in 2016. Results from HRM-PCR-based assignment to ST8 or ST6 show that the number of cases of MRSA t304/ST8 was high in the initial years of the study period (Fig. 1), but decreased steadily until 2011. The situation was reversed for MRSA t304/ST6, with a sharp increase in cases from 2008 to 2016 (Fig. 1). Of the total MRSA t304 strains, 318 (66.8%) were included for further analyses.

### Epidemiology

**Fig. 2:**
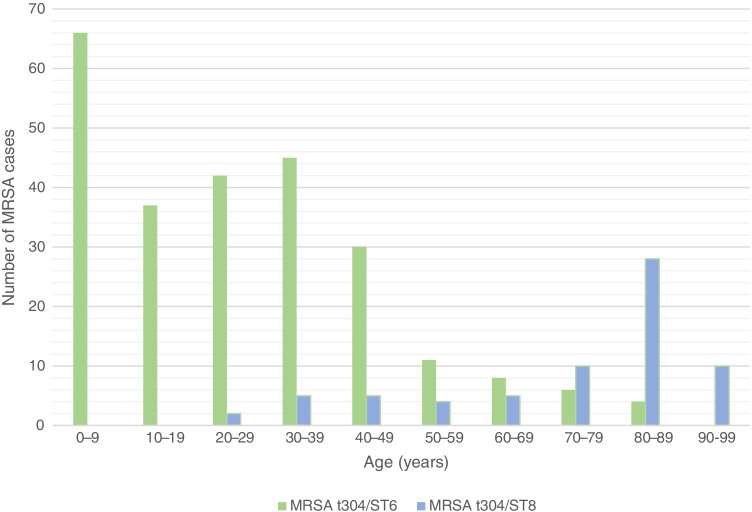
Age distribution of patients with MRSA t304/ST8 (*n* = 69) and t304/ST6 (*n* = 249)

The epidemiological data revealed a significant distinction between the age distributions of the MRSA t304/ST8 and MRSA t304/ST6 strains (Table 1, Fig. 2). The average age was 73 years (range 23–97 years) for persons with t304/ST8, compared to 26 years (range 0–85 years) for t304/ST6. In the t304/ST8 group, there were more women (65.2%) than men (34.8%).

**Table 1:** Clinical and epidemiological characteristics of MRSA t304 cases

	**Category**		**t304/ST8**		**t304/ST6**		***P*-value**^**3**^
			**n**	**%**	**n**	**%**	
**Total**	Cases		69	21.7	249	78.3	
**Age and sex**	Age, average		73		26		
	Age, range		23–97		0–85		**1.76E-33**
	Fraction of men		24	34.8	125	50.2	**4.7E-02**
	Fraction of women		45	65.2	124	49.8	
**Birth country**	Norway		57	82.6	96	38.6	**2.7E-11**
	Norwegian heritage		57	100.0	50	52.1	**6.6E-12**
	Non-Norwegian heritage^1^		0	0.0	46	18.5	
	Non-Norwegian birth country		11	15.9	153	61.4	**2.7E-11**
	Africa except Egypt		2	18.2	24	15.7	**1.2E-04**
	Asia except Middle East^2^		5	45.5	38	24.8	
	Europe except Norway		4	36.4	8	5.2	
	Middle East		0	0.0	81	52.9	
	Unknown/other		1	9.1	2	1.3	
**MRSA acquired**	Norway		51	73.9	42	16.9	**3.8E-21**
	Abroad		1	1.4	115	46.2	
	Work-related		0	0.0	3	2.6	1.6E-01
	Home country visit		0	0.0	31	27.0	
	Tourism		1	100.0	8	7.0	
	Immigration		0	0.0	65	56.5	
	Unknown		17	24.6	92	36.9	
**Carriage/infection**	Carriage		51	73.9	188	75.5	9.5E-01
	Infections		18	26.1	61	24.5	
	Wound/pus		16	88.9	49	80.3	6.0E-01
	Other infections		2	11.1	12	19.7	
**Outbreaks**	Outbreak-related cases		46	66.7	2	0.8	**1.9E-34**
	Non-outbreak-related cases		23	33.3	247	99.2	
**HA/CA**	HA-MRSA		62	89.9	62	24.9	**1.3E-22**
	Infections		15	24.2	15	24.2	1.0E + 00
	Carriage		47	75.8	47	75.8	
	CA-MRSA		7	10.1	187	75.1	**1.3E-22**
	Infections		3	42.9	46	24.6	4.8E-01
	Carriage		4	57.1	141	75.4	

^1^Defined as a case where at least one parent was born abroad

^2^Definition of the Middle Eastern Countries according to World Population Review: Bahrain, Cyprus, Egypt, Iran, Iraq, Israel, Jordan, Kuwait, Lebanon, Oman, Palestine, Qatar, Saudi Arabia, Syria, Turkey, The United Arab Emirates, and Yemen

^3^Corrected *p*-values, significant values displayed in bold. For birth country, the *p*-value corresponds to comparison between Norway and non-Norwegian birth country; for HA/CA, the given *p*-value corresponds to comparison between HA-MRSA and CA-MRSA

The registered place of acquisition for MRSA cases also differed distinctly between the ST8 and ST6 groups (Table 1). One single case of MRSA t304/ST8 was reported as contracted abroad; the corresponding number for ST6 cases was 115/249 (46.2%). Among these, a majority were associated with either immigration (65/115, 56.5%) or home country visit (31/115, 27.0%). Only 8 ST6 cases were connected to tourism, and 3 were work-related.

**Fig. 3:**
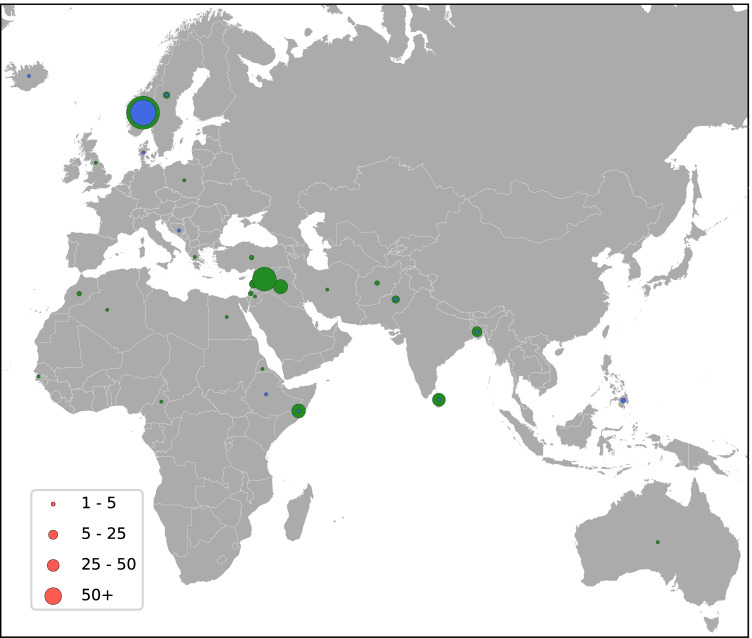
Map displaying birth country for patients with MRSA t304/ST8 (blue) and t304/ST6 (green), with circles sized relative to the number of cases in each country

There was a significant difference between the ST8 and ST6 groups in terms of association to birth country (Table 1, Fig. 3). In the ST8 group, 57/69 (82.6%) persons were born in Norway to Norwegian parents. In the ST6 group, 153 (61.4%) were born abroad. Of these, 81 (52.9%) were born in Middle Eastern countries, most frequently in Syria and Iraq, while 38/153 (24.8%) persons were born in other Asian countries. Forty-six persons (18.5%) with ST6 had non-Norwegian heritage with at least one parent born abroad, 41 of these 46 were children from 0 to 18 years.

### Clinical characteristics

The infection rates of MRSA t304/ST8 and t304/ST6 were similar, 26.1% in the ST8 group and 24.5% in the ST6 group (Table 1). Wound infections and abscesses were the dominating causes of infections in both groups, causing 88.9% of the infections in the ST8 group and 80.3% in the ST6 group. Other infections (*n* = 14 for ST8 and ST6) included bacteremia, pneumonia, genital infection, mastitis, media otitis, conjunctivitis, and respiratory infection of unknown significance. Only 14/318 (4.4%) persons with infections were admitted to hospital at the time the MRSA strains were collected.

**Fig. 4:**
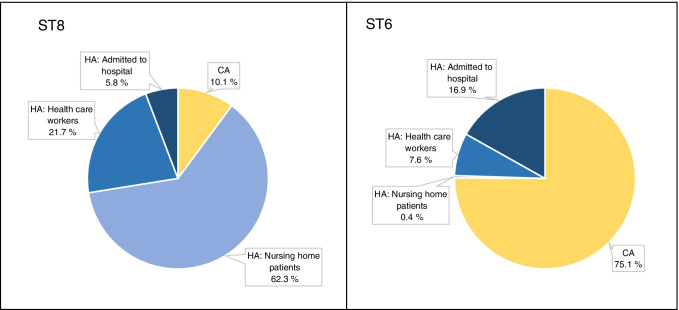
Epidemiological characteristics of MRSA t304 cases. Comparison of cases defined as community-associated or healthcare-associated for t304/ST8 and t304/ST6 cases

In total, 62 of the 69 (89.9%) t304/ST8 cases were defined as HA-MRSA (Table 1). This included 43 (62.3%) persons living in a nursing home, 15 (21.7%) HCWs, and 4 (5.8%) persons admitted to a hospital (Fig. 4). The infection rate within t304/ST8 HA-MRSA was 24.2%.

Sixty-two (24.9%) of the t304/ST6 cases were defined as HA-MRSA (Fig. 4). Only one person lived in a nursing home, 19 (7.6%) were HCWs, and 42 (16.9%) were admitted to hospital. CA-MRSA accounted for 187/249 (75.1%) of the ST6 group. The infection rate was 24.2% for HA-MRSA and 24.6% for CA-MRSA.

Forty-eight (15.1%) of the MRSA t304 strains were reported to be outbreak related; almost all these were t304/ST8 (46/48) (Table 1). The majority of outbreak-related cases included persons living in nursing homes (35/48, 72.9%) belonging to 6 different outbreaks, where the largest outbreak included 21 persons.

### Antimicrobial susceptibility

**Fig. 5:**
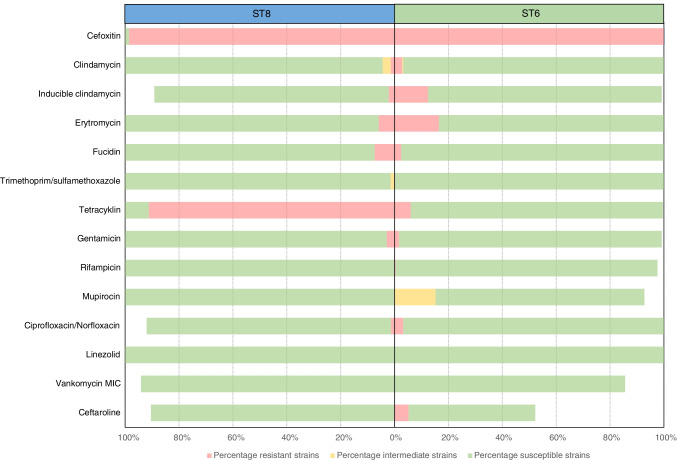
Phenotypic antimicrobial susceptibility of MRSA t304/ST8 and t304/ST6 strains

Both sequence types of MRSA t304 displayed limited antimicrobial resistance, as 59.1% of all strains were resistant only to cefoxitin/betalactams (Fig. 5). Resistance against two antibiotic groups, including cefoxitin, was detected in 111 strains (34.9%), 16 strains (5.0%) were resistant against 3 antibiotic groups, and only 3 strains (0.9%) displayed resistance against 4 or 5 different antibiotic groups. The t304/ST8 group had higher occurrence of tetracyclin resistance (91.2%) compared to the t304/ST6 group (6.0%).

### Resistance and virulence genes

**Fig. 6:**
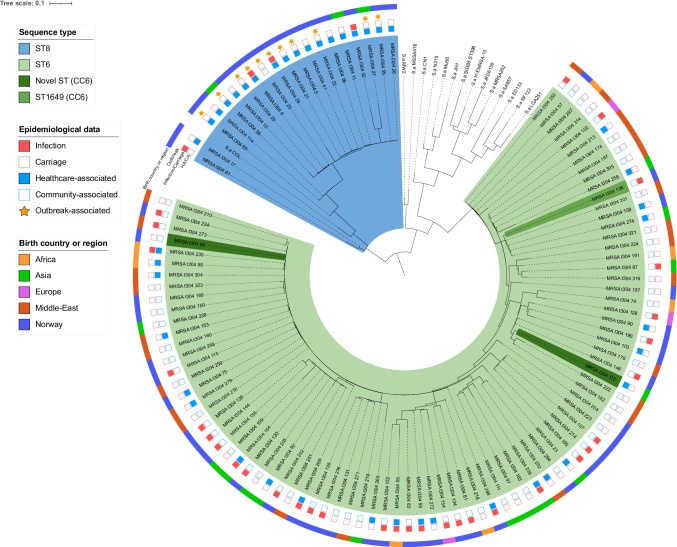
Pan-genome phylogeny of MRSA t304/ST8 and t304/ST6 with epidemiological data and birth country

WGS was performed on 20 t304/ST8 strains and 88 t304/ST6 strains. Phylogenetic analysis of the pangenome (Fig. 6) showed that the two groups were clearly distinct lineages, with relatively low core genome SNP distances within each group (median 26 SNPs and 62 SNPs for MRSA t304/ST8 and t304/ST8 accordingly). Three strains had point mutations in MLST alleles (other than *pta*) causing novel (*n* = 2) or other (*n* = 1) sequences types within CC6. In general, infection strains and carriage strains were dispersed, and there were no clear correlations between specific clusters and country of birth.

Results from in silico resistance gene prediction showed low number of resistance genes for both groups of MRSA t304 (Figure S1). All strains shared *mecA*, *fosB*, and *tet(38)*. Among the t304/ST8 group, 18/20 (90.0%) strains had *tetM*, 17/20 (85.0%) *blaZ*, and 17/20 (85%) *dfrC*. The t304/ST6 strains displayed even lower occurrence of resistance genes, with 16/88 (18.2%) *ermC*, 5/88 (5.7%) *tetK*, and 3/88 (3.4%) *blaZ*.

There were 72 different virulence factors detected in the sequenced MRSA t304 strains (*n* = 108) (Figure S1), of which 46 were present in all strains and 11 in more than 94% of strains. On average, 64 and 67 virulence factors were found in t304/ST8 and t304/ST6, respectively. The main differences included a complete serotype 8 capsule gene cluster in t304/ST6, which was not detected in the t304/ST8 strains. Most t304/ST6 strains (90.0%) encoded staphylococcal enterotoxin D, which was not found in any of the t304/ST8 strains. Furthermore, the MSCRAMMs encoded by *clfB*, *cna*, *sdrC*, and *sdrE* were found in a majority of t304/ST6 strains (> 68.5%), while only in a minority of t304/ST8 strains (< 10.0%).

In silico SCC*mec* typing revealed that SCC*mec* type IV or IVa dominated in both sequence types, while a small subset of t304/ST6 strains had SCC*mec* type IVc (*n* = 6) or SCC*mec* type V (*n* = 4) (Figure S1).

## Discussion

Two different MRSA t304 clones have been circulating in Norway, detected in different epidemiological settings. While t304/ST8 was already established in Norway before the study period [4, 23] and has not been reported since 2014, t304/ST6 has caused a sharp increase of MRSA t304 in recent years. Two different clones sharing the same *spa* type appears to be a rare event [24], but this study is still a reminder that spa typing may have insufficient discriminatory power for surveillance of MRSA, even in the epidemiologically heterogeneous MRSA situation in Norway.

The MRSA t304/ST8 clone showed a significant association with outbreaks in nursing homes, with many cases among elderly persons and HCWs, which explains the female dominance among the ST8 cases. Due to this, t304/ST8 was initially designated as healthcare-associated, but our study finds little evidence of this clone being particularly adapted to the HA setting. Rather, based on SCCmec type, minimal antimicrobial resistance as well as high rate of carriage, it displays many typical CA-MRSA hallmarks, except for the absence of PVL. No detected cases of t304/ST8 since 2014 could be an effect of good handling of outbreaks and/or improved infection control measures in nursing homes during the recent years.

The MRSA t304/ST6 clone was mainly community-associated, mostly detected in younger people and children. A large proportion of cases were either acquired abroad following immigration or home-country visit and/or had non-Norwegian heritage or birth country. Only a small number of cases were associated with work-related travel or vacation, indicating that transmission of this clone may require close contact with the local population. A majority of the non-Norwegian born persons with MRSA t304/ST6 were from Syria and Iraq, indicating a Middle Eastern origin of this clone, as was previously also suggested in a Danish study [25]. The sharp increase of MRSA t304/ST6 in Norway during the study period may partly be a result of increased immigration from these countries due to the refugee crisis which peaked in 2015–2016. MRSA t304/ST6 was also acquired and/or detected in significant numbers in persons born in Africa and other Asian countries, suggesting repeated introductions of this clone to Norway during the study period. Despite this, phylogenetic analysis displayed a surprisingly conserved t304/ST6 clone, with very little variation in terms of resistance and virulence genes.

The limitations of this study include incomplete information from the registers used, for example, on the place of acquisition of MRSA. A significant number of cases did furthermore not have available address information, particularly immigrants, and excluding these persons from the study may have led to an underestimation of the number of imported cases. On the other hand, more comprehensive screening routines among refugees may potentially have contributed to higher numbers of detected MRSA cases in this group. Higher screening rates around HA-related MRSA cases than CA-related cases are also likely, leading to an overestimation of HA-related cases. The broad definition of HA-MRSA used in this study may also be debated, since the place MRSA was diagnosed is not necessarily where MRSA was acquired. Information about the time of admission to healthcare institutions would probably have reduced the number of HA-MRSA, providing the ability to exclude MRSA detected within 48 h of admission.

The main aim of the study was to compare the two different MRSA clones and determine whether MRSA t304/ST6 represents a high risk clone, which would warrant closer attention in our national surveillance program. Our results, however, show that MRSA t304/ST6 does not appear to be especially virulent, with low levels of antibiotic resistance and mainly associated with carriage in the community. As far as we know, MRSA t304/ST6 has not yet caused outbreaks in Norwegian healthcare institutions. However, this needs to be carefully monitored, since most of the MRSA-positive persons are still young and presumably healthy. If many become persistent carriers of MRSA, this situation could change with time. There is special concern about vulnerable patients in, e.g., neonatal units, where many MRSA outbreaks have taken place the recent years, including one caused by MRSA t304/ST6 [7, 26, 27]. Active surveillance of MRSA and characterization of emerging MRSA clones, as performed in this study, is thus important in order to evaluate and potentially adjust existing MRSA screening and infection control guidelines to prevent MRSA from establishing in the Norwegian healthcare system.

## Supplementary Information

Below is the link to the electronic supplementary material.

Supplementary file1 Pan-genome phylogeny of MRSA t304/ST8 and t304/ST6 with SCCmec type, resistance- and virulence genes (PDF 68 KB)

## Data Availability

Due to Norwegian legislation on medical and health research, the full clinical and epidemiological dataset cannot be made openly available as per ethical approval by the Regional Committees for Medical and Health Research Ethics (REC). Sequence data are available from GenBank under BioProject ID PRJNA737487.
