# Supplementary figures and images for: A tale of two STs: molecular and clinical epidemiology of MRSA t304 in Norway 2008–2016
Source: Eur J Clin Microbiol Infect Dis. 2021 Oct 23;41(2):209–18. doi: 10.1007/s10096-021-04353-9 (PMC8770451; doi:10.1007/s10096-021-04353-9)

scale: 0.1

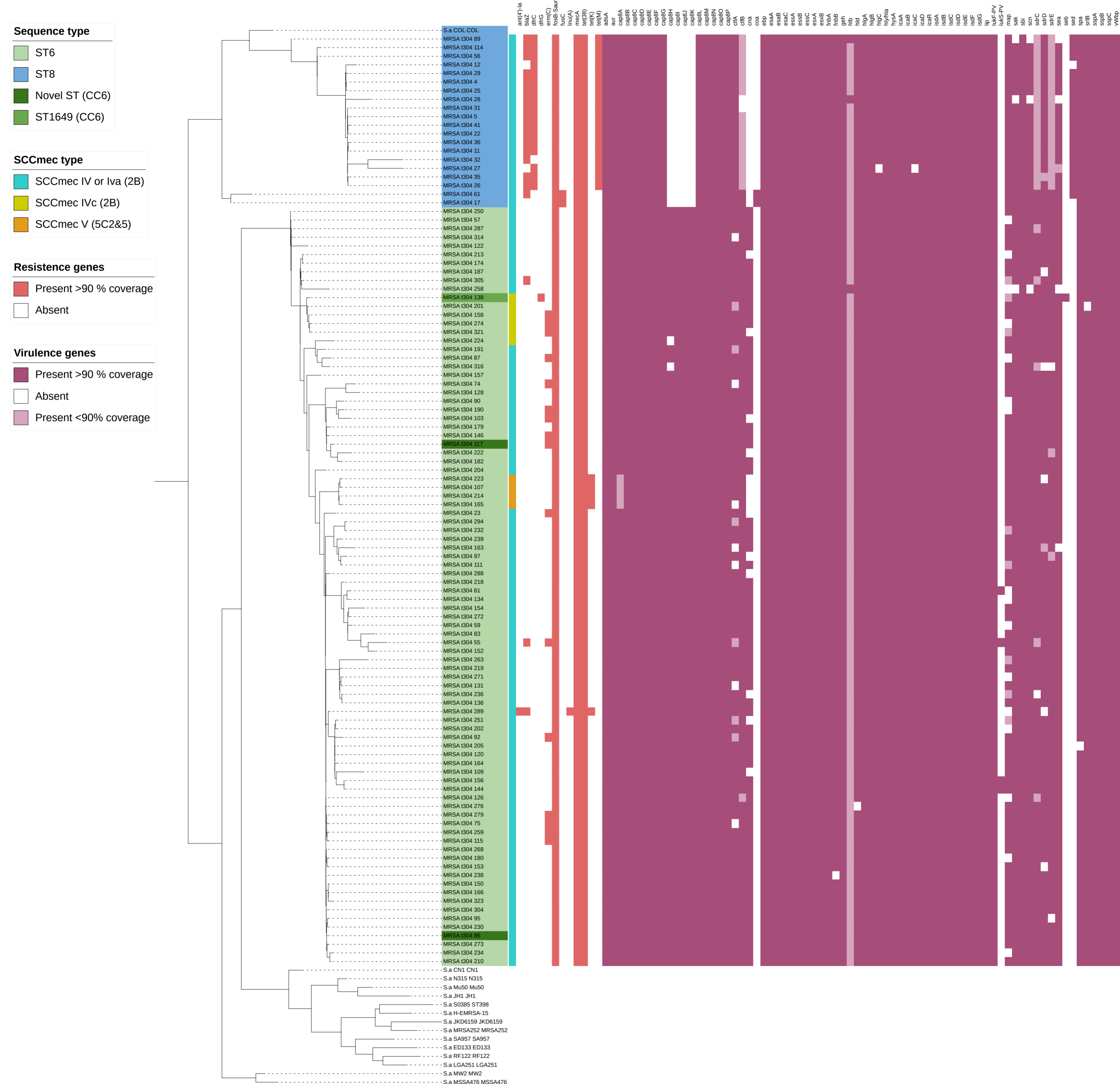

Supplement: Supplementary file 1 — Pan-genome phylogeny of MRSA t304/ST8 and t304/ST6 with SCCmec type, resistance- and virulence genes (PDF 68 KB) [file 10096_2021_4353_MOESM1_ESM.pdf]
